# Analysis of *Ureaplasma urealyticum*, *Chlamydia trachomatis*, *Mycoplasma genitalium* and *Neisseria gonorrhoeae* infections among obstetrics and gynecological outpatients in southwest China: a retrospective study

**DOI:** 10.1186/s12879-021-06966-z

**Published:** 2022-03-25

**Authors:** Ting Liu, Shu-yu Lai, Wei Zhou, Yan-ling Liu, Sha-sha Chen, Yong-mei Jiang

**Affiliations:** 1 Department of Laboratory Medicine, West China Second University Hospital, Sichuan University, No. 20, Section 3, Renmin South Road, Chengdu, Sichuan 610041 People’s Republic of China; 2 Key Laboratory of Obstetric and Gynecologic and Pediatric Diseases and Birth Defects of Ministry of Education, Sichuan University, Chengdu, People’s Republic of China; 3 State Key Laboratory of Biotherapy and Cancer Center/National Collaborative Innovation Center for Biotherapy, Sichuan University, Chengdu, China; 4 Department of Laboratory Medicine, The First Hospital of Lanzhou University, Lanzhou, Gansu China

**Keywords:** *Ureaplasma urealyticum*, *Chlamydia trachomatis*, *Mycoplasma genitalium*, *Neisseria gonorrhoeae*, Reproductive tract infection

## Abstract

**Background:**

The aim of this study was to analyze the present situation of *Ureaplasma urealyticum* (UU), *Chlamydia trachomatis* (CT), *Mycoplasma genitalium* (MG) and *Neisseria gonorrhoeae* (NG) infections among obstetrics and gynecological outpatients in southwest China.

**Methods:**

A total of 3225 urogenital swabs were included in this study. All swabs were tested by RNA-based simultaneous amplification and testing (SAT) methods. Routine analysis of leucorrhea smear and drug susceptibility were performed in UU positive patients.

**Results:**

Of these 3225 outpatients, the positive rate was 27.07% for UU, 4.99% for CT, 3.10% for MG, and 0.09% for NG. UU, CT, and MG infections were more common in women of reproductive age (aged 25–34 years), while NG infection was more prominent in women aged 30–34 years and over 40 years. Overall, the infection rate of UU was significantly higher than that of the other three infections, and UU also played a major role even in the mixed infections. 65.07% of the UU positive patients had negative results on routine leucorrhea smear analysis, and the remaining patients may have bacterial vaginitis (15.79%), fungal vaginitis (11.48%), trichomonas vaginitis (0.96%) or other vaginal inflammation (6.70%). We have observed that maternal UU infection can lead to low birth weight, neonatal pneumonia, and premature delivery. The results of the drug susceptibility test of UU showed a higher sensitivity to pristinamycin, doxycycline, tetracycline, clarithromycin, and josamycin (100%, 97.0%, 96.4%, 95.9%, and 95.3%, respectively), and lower sensitivity to ciprofloxacin and ofloxacin (2.4% and 4.7% respectively).

**Conclusions:**

This study could contribute to a better understanding of the current epidemiological features of UU, CT, MG, and NG among obstetrics and gynecological outpatients in southwest China, and thus facilitate to development of the more effective intervention, prevention, and treatment of reproductive tract infection.

## Introduction

*Ureaplasma urealyticum* (UU), *Chlamydia trachomatis* (CT), *Mycoplasma genitalium* (MG), and *Neisseria gonorrhoeae* (NG) are the common sexually transmitted pathogens of the reproductive tract. Previous studies have found that these pathogen infections are closely related to some gynecological diseases, such as non-gonococcal urethritis, vaginitis, cervicitis, endometritis, and even infertility [1, 2]. Generally, UU, CT, MG, and NG exist in epithelial cells and mostly do not invade the blood. UU belongs to the ureaplasma group with a circular double-stranded DNA genome. It is the smallest and simplest cell capable of self-reproduction. In recent years, UU is one of the main pathogens that cause non-gonococcal urethritis, accounting for approximately 70% of all cases [3, 4]. It’s worth noting that UU infections tends to occur in young people, especially after unclean sexual intercourse, resulting in infertility, abortion and fetal growth retardation, and other fertility problems in women of childbearing age [5, 6]. CT is an intracellular parasite that contains DNA and RNA, with an infection rate of 9.8% [7, 8]. It has a variety of serotypes, and different serotypes may cause different clinical manifestations in humans [9]. MG has the least genomic of all mycoplasmas. It also causes non-gonococcal urethritis and inflammation of the cervix or endometrium in women, with an infection rate of up to 25% [10]. NG is a gram-negative diplococcus. The main clinical manifestations of NG infection are urethritis and cervicitis [9]. Among the above four pathogens, NG is the only one that can be observed under the microscope by gram staining [11]. The clinical manifestations of patients with UU, CT, MG, and CT infections are varied, and even some patients have no obvious clinical symptoms. Therefore, a better understanding of the infection status and characteristics of these pathogens is necessary.

Simultaneous amplification and testing (SAT) is a molecular diagnostic method based on isothermal amplification of pathogen RNA, which can accurately and rapidly detect a variety of pathogens [12]. The application of SAT in the detection of RNA of pathogens of sexually transmitted diseases can overcome the disadvantages of traditional microbial culture methods. SAT method is more time-saving and more sensitive. Besides, SAT test targets the RNA of pathogens, which adequately reflects a patient’s infection status and avoids antibiotic overuse. In this study, we included 3225 outpatients in the department of obstetrics and gynecology to evaluate the prevalence characteristics of UU, CT, MG, and NG in the southwest of China using the SAT method. A better understanding of UU, CT, MG, and NG infection can help policymakers identify and address their specific strategy for the prevention, testing, and treatment of these infections.

## Materials and methods

### Patients

A total of 3225 outpatients who visited the obstetrics and gynecology department of West China Second University Hospital (Chengdu, China) from 2019 to 2021 for various reasons were included in this study. Urogenital swabs were collected and further detected the status of the infection of UU, CT, MG, and NG with SAT method. The average age of the patients was 31 years (range from 16 to 75 years), and the inclusion criteria were as follows: (a) Women who have no other serious diseases, such as diabetes, hypertension, kidney diseases, etc. (b) Women who have no abnormalities in uterus and ovary by B-ultrasonography examination. (c) Women who underwent UU, CT, and MG screening for the first time in the past 12 months. The exclusion criteria were as follows: (a) Women who have had vaginal medication in the past 2 weeks. (b) Women who have had antibiotics in the past 2 weeks. (c) Women who could not participate in this study for any reason. All the inspections were performed in the Department of Laboratory Medicine in West China Second University Hospital.

### Specimen collection

Urogenital swabs sampling was performed by outpatient physicians. For SAT experiments, all samples were placed into the preservation solution in time according to the operational guidelines to prevent the degradation of the RNA of pathogenic microorganisms, stored at 2–8 °C and timely tested within 24 h using commercial kits (Shanghai Rendu Biotechnology Co., Ltd). Routine analysis of leucorrhea was performed by an automatic gynecological secretion analysis system (GMD-S600, DI RUI, Made in China), and drug susceptibility was performed by Mycoplasma IST 2 (bioMerieux, SA, French).

### Simultaneous amplification and testing (SAT)

The SAT experiment is briefly divided into two steps, RNA extraction and amplification, using commercial kits purchased from Rendu Biotechnology Co., Ltd (Shanghai, China). RNA extraction was performed by magnetic beads methods and eluted with 40 μl detection reagent (PCR buffer, dNTP, NTP, 500 nM each of primers, 250 nM of optimization probes and internal control). For amplification, 30 μl RNA sample and 10 μl enzyme reagent (M-MLV reverse transcriptase and T7 RNA polymerase) were mixed as 40 μl final detection system. All reactions were run on an ABI 7500 detection system (Life Technologies, CA, USA) in following conditions: 40 cycles of 42 °C for 60 s. Two fluorescent dyes of FAM and VIC were used to detect the target gene and internal control respectively. Results were considered positive when a clear melting curve of the expected site was obtained (dt ≤ 35, Tm = 68 ± 5 °C).

### Drug susceptibility analysis

For this experiment a commercial kit, Mycoplasma IST-2 (BioMerieux, SA, France), was used according to the manufacturer’s instructions. The kit contains strips that give information on the presence or absence of UU and also provide additional information on antibiotic susceptibility to doxycycline, josamycin, ofloxacin, erythromycin, tetracycline, ciprofloxacin, azithromycin, clarithromycin and pristinamycin. Briefly, a urogenital swab specimen was inoculated into 3 ml R1 solution (transport medium), and then combined with a vial of R2 (lyophilized powder). A 55 μL aliquot of the above solution was dispensed into each of 22 reaction wells on the reagent strip. The strips were incubated at 36 ± 2° C for 24 and 48 h. A positive test was indicated by a change in broth color from yellow to red (cut-off value is 104 color-changing units per ml).

### Statistical analysis

Frequency and percentage were analyzed with SPSS 20.0 statistical software in this study. Student’s t-test and Chi-square test were used to compare categorical variables. Differences were considered to be statistically significant when p < 0.05.

## Results

### Prevalence of UU, CT, MG, and NG infections among the study population

**Fig. 1:**
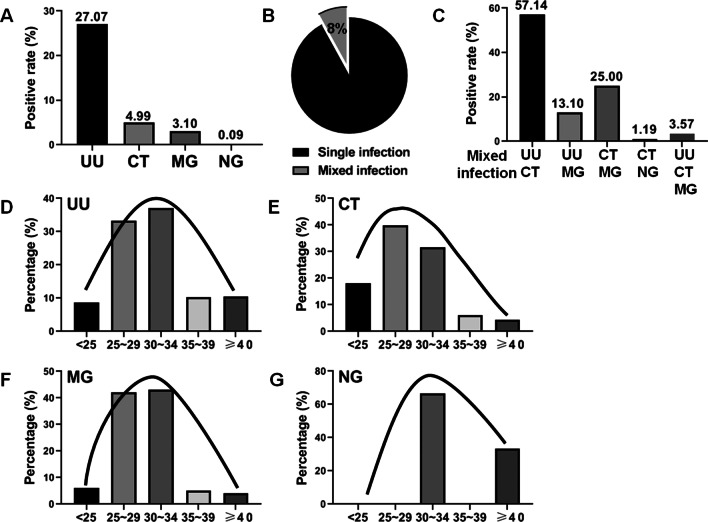
The prevalence of UU, CT, MG, and NG infections among the study population. **A** total of 3225 urogenital swabs were collected to detect UU, CT, MG, and NG infections by SAT. **A** The positive rates of UU, CT, MG, and NG in the included population were 27.07% (873/3225), 4.99% (161/3225), 3.10% (100/3225) and 0.09% (3/3225), respectively. **B**, **C** Among all the positive infection cases, 84 cases (84/1050, 8.00%) were found to be mixed infections, including UU + CT, UU + MG, CT + MG, CT + NG and UU + CT + MG. **D**–**G** For the age prevalence, UU, CT, and MG infections were more common in women of reproductive age (aged 25–34 years), while NG infection was more prominent in women aged 30–34 years and over 40 years. These data suggest that the infection rate of UU was significantly higher than that of the other three infections, and UU also played a major role even in the mixed infections. Compared with NG infection, UU, CT and MG infections are more common in young women

Of these 3225 outpatients, a total of 873 patients were positive for UU (873/3225, 27.07%), 161 patients were positive for CT (161/3225, 4.99%), 100 patients were positive for MG (100/3225, 3.10%) and three patients were positive for NG (3/3225, 0.09%) (Fig. 1A). Among all the positive infection cases, 84 cases (84/1050, 8.00%) were found to be mixed infections, including UU + CT, UU + MG, CT + MG, CT + NG, and UU + CT + MG (Fig. 1B and C). More than half of the mixed infections were UU + CT (48/84, 57.14%). UU, CT, and MG infections were more common in women of reproductive age (aged 25–34 years), while NG infection was more prominent in women aged 30–34 years and over 40 years (Fig. 1D–G). Overall, the infection rate of UU was significantly higher than that of the other three infections, and UU also played a major role even in the mixed infections.

### Clinical manifestations of UU, CT, MG, and NG infections

**Fig. 2:**
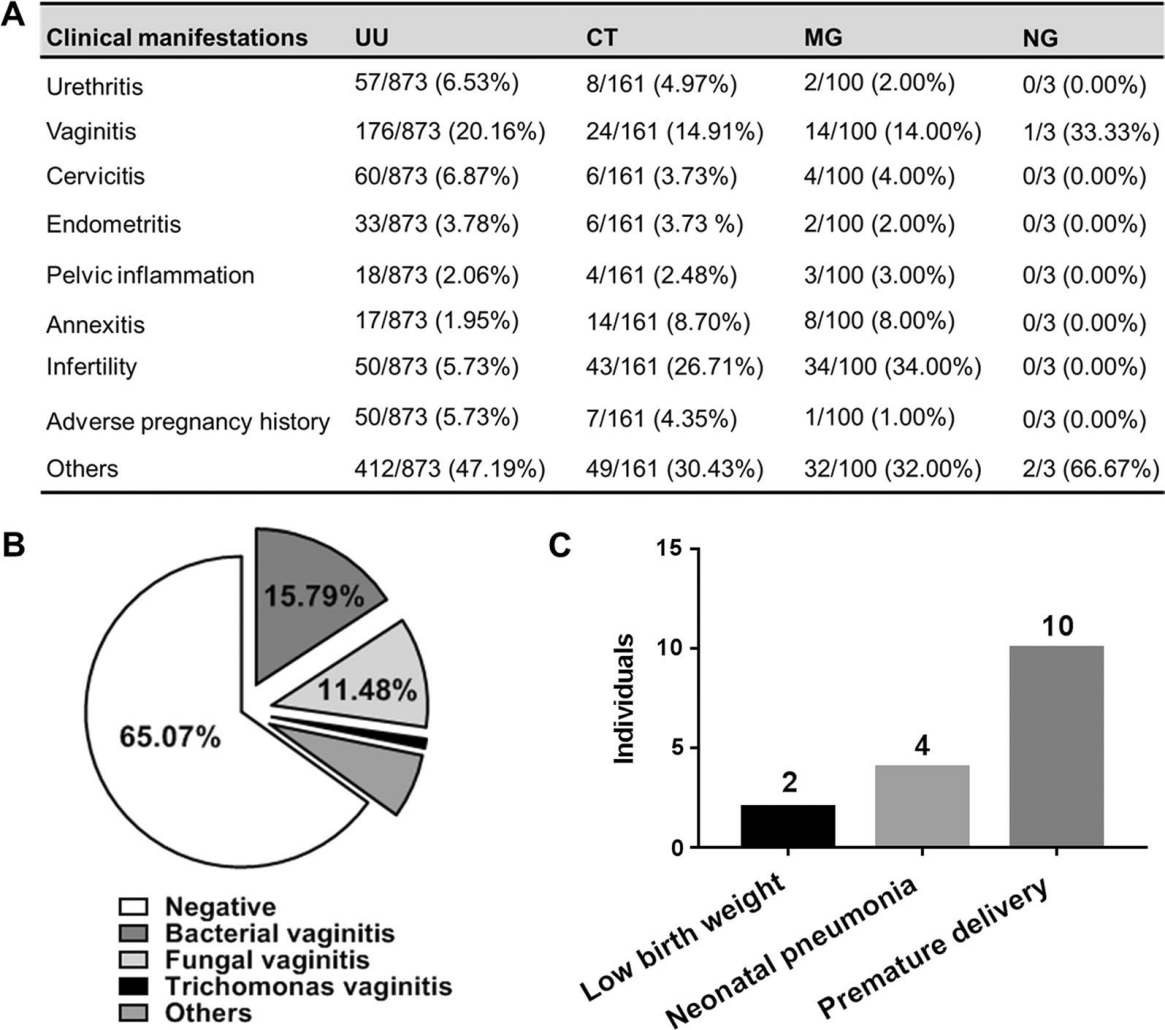
Clinical manifestations of UU, CT, MG, and NG infections. **A** The most common clinical manifestations in patients with UU, CT, MG, and NG infections were vaginitis, cervicitis, urethritis and annexitis. In addition, the proportion of infertility patients and patients with adverse pregnancy history was relatively high. About 30 ~ 40% of patients had no clinical symptoms. **B** Leucorrhea analysis was performed in 209 UU positive patients. Only 34.93% of UU positive patients had vaginal inflammation, of which 15.79% were bacterial vaginitis, 11.48% were fungal vaginitis, 0.96% were trichomonas vaginitis and 6.70% for other vaginal inflammation (WBC > 15/HPF). **C** Of all 873 UU positive cases followed, we observed 10 cases of premature delivery, 2 cases of low birth weight, and 4 cases of neonatal pneumonia associated with maternal UU infection. These results suggest that not all patients with UU infection have significant clinical symptoms. Therefore, screening for urogenital infections in pre-pregnant women without any clinical symptoms is still necessary to avoid adverse pregnancy outcomes

As shown in Fig. 2A, the most common clinical manifestations in UU positive patients were vaginitis (176/873, 20.16%), cervicitis (60/873, 6.87%) and urethritis (57/873, 6.53%). While the most common clinical manifestations of CT and MG positive patients were vaginitis (14.91% and 14.00%, respectively) and annexitis (8.70% and 8.00%, respectively). It is worth mentioning that in UU, CT and MG positive patients, the proportion of infertility patients and patients with adverse pregnancy history was relatively high. In addition, 30 ~ 40% of patients had no clinical symptoms and were found to have latent infections through routine annual gynecological examination or routine prenatal examination. For the leucorrhea analysis of UU positive patients in Fig. 2B, 65.07% of patients had normal results (negative), and the remaining patients had 15.79% for bacterial vaginitis, 11.48% for fungal vaginitis, 0.96% for trichomonas vaginitis, and 6.70% for other vaginal inflammation (WBC > 15/HPF and no fungi, trichomonas, and clue cells were found). We have also observed that maternal UU infection can lead to premature delivery (10 cases), low birth weight (2 cases), and neonatal pneumonia (4 cases) of all the 873 patients with UU infection (Fig. 2C). Overall, more than half of women with UU infection have no clinical symptoms, and about 35% of patients may have vaginal inflammation. Furthermore, as shown in Fig. 1A, the positive rate of UU is very high in outpatient patients of obstetrics and gynecology, which is about 1/4 of all patients. Therefore, routine detection of UU infection in women before pregnancy is very necessary to effectively avoid the occurrence of UU related premature delivery, low birth weight, and neonatal pneumonia.

### Drug susceptibility analysis of *Ureaplasma urealyticum*

As shown in Table 1, a total of 169 UU samples were used to detect drug sensitivity of 9 antibodies, including ciprofloxacin (CIP), azithromycin (AZM), clarithromycin (CLR), erythromycin (ERY), josamycin (JOS), doxycycline (DOX), ofloxacin (OFX) pristinamycin (PRI) and tetracycline (TCY). The results of the drug susceptibility test of UU showed a higher sensitivity to pristinamycin, doxycycline, tetracycline, clarithromycin, and josamycin (100%, 97.0%, 96.4%, 95.9%, and 95.3%, respectively), and lower sensitivity to ciprofloxacin and ofloxacin (2.4% and 4.7% respectively). Overall, UU samples collected in this study are sensitive to tetracyclines and macrolides, and timely use of antibiotics can effectively treat UU infection in the clinic.

## Discussion

The aim of this study was to detect UU, CT, MG, and NG infections in urogenital swabs from outpatients of obstetrics and gynecology in southwest China by SAT method. These findings would contribute to a better understanding of the current epidemiological features of UU, CT, MG, and NG among obstetrics and gynecological outpatients in southwest China and thus facilitate to develop a more effective intervention, prevention, and treatment of reproductive tract infection. As shown above, the infection rate of UU was significantly higher than that of the CT, MG, and NG infections and UU also played a major role even in the mixed infections. For the prevalence of UU, CT, MG, and NG in different age groups, UU, CT, and MG infections were more common in women of reproductive age (aged 25–34 years), while NG infection was more prominent in women aged 30–34 years and over 40 years. In addition, maternal UU infection may lead to premature delivery, low birth weight, and neonatal pneumonia, but not all patients with UU infection have obvious clinical manifestations [13]. These results are consistent with previous studies [5, 6, 14]. In this study, women with UU positive pregnancies were tracked, among which ten were premature, 2 were low weight, and 4 were neonatal pneumonia (As shown in Fig. 2). As for abortion, some studies found that the positive rate of UU was as high as 84.4% from the aborted tissues [13]. The perinatal infection caused by UU has become a new problem in modern obstetrics. Therefore, the SAT test is necessary for women before pregnancy. In the study of antibiotic sensitivity of UU, we found that UU was sensitive to pristinamycin, doxycycline, tetracycline, clarithromycin, and josamycin, with over 90% of all samples tested being susceptibility to these antibodies.

Additionally, in this study, the positive rate of NG was very low; this might be due to the fact that a large proportion of NG has been detected in routine leucorrhea microscopy tests. However, UU, CT, and MG are difficult to be diagnosed by routine microscopic examination of vaginal or cervical secretions. Mycoplasma and chlamydia are also difficult to be identified by microbial culture due to their slow growth and high nutrient requirements [15, 16]. In particular, the culture of MG in clinical specimens is more difficult to succeed [17]. SAT method is designed to amplify 16S rRNA in cells, thus providing a more sensitive and faster method for the detection of pathogens, such as mycobacterium tuberculosis complex, enterovirus 71, UU, CT, NG, and MG [15, 18–20]. As we can see in Fig. 2 that urogenital tract infections such as UU are likely to be asymptomatic in most patients. When the immune system is weakened, or a woman is pregnant, UU infection may cause inflammation of the female reproductive system. It may also spread through the placenta into the uterine cavity and cause adverse consequences such as abortion, premature delivery or low birth weight due to the increase of progesterone and the suppression of cellular immunity. Currently, there are no vaccines related to other genitourinary pathogens other than HPV, and the research and development of the vaccine is an area worth looking forward to [21]. In general, screening for UU, CT, MG, and NG before pregnancy is important even for women without clinical symptoms to effectively promote the early diagnosis and treatment of these infections, and to avoid adverse pregnancy outcomes.

The study still has some limitations. Firstly, all the experiments were conducted at the West China Second University Hospital, which is the largest women’s and children’s hospital in southwest China, but a larger study in multiple centers may make the data more convincing. Secondly, due to the limitations of retrospective studies, the sample size of the patients with UU infection and drug susceptibility analysis is small, so it is necessary further to increase the sample size for analysis in future studies. Finally, although we can provide data on the prevalence of infections, it is difficult to draw any conclusions about the role or the mechanisms of these infections in pregnancy outcomes. Further research will be carried out on this part in the future.

## Conclusion

Our study retrospectively analyzed the prevalence of UU, CT, MG, and NG infections among obstetrics and gynecological outpatients in southwest China and tracked the pregnancy outcomes in women with UU infection. Considering high prevalence of UU among the population in southwest China, urogenital infection screening is recommended in symptomatic patients with suspected urogenital infections and women pre-pregnancy. In conclusion, our study could contribute to a better understanding of the current epidemiological features of UU, CT, MG, and NG among obstetrics and gynecological outpatients in southwest China, and thus facilitate to development of more effective intervention, prevention, and treatment of urogenital infections.

**Table 1:**
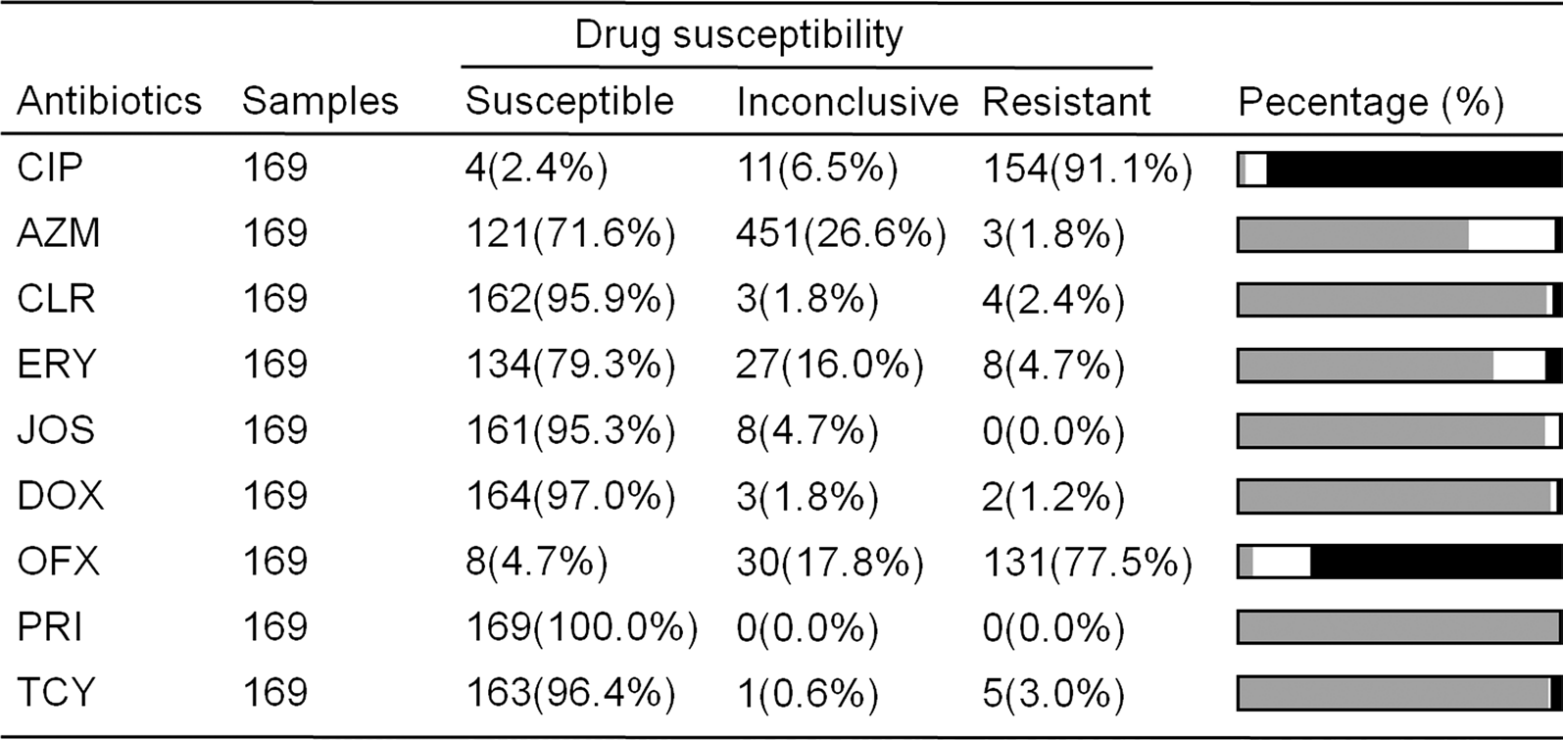
Drug susceptibility analysis of UU

*CIP* Ciprofloxacin, *AZM* Azithromycin, *CLR* Clarithromycin, *ERY* Erythromycin, *JOS* Josamycin, *DOX* Doxycycline, *OFX* Ofloxacin, *PRI* Pristinamycin, *TCY* Tetracycline

## Data Availability

All data generated or analyzed during this study are included in this published article.

## Abbreviations

UU: *Ureaplasma urealyticum*
CT: *Chlamydia trachomatis*
MG: *Mycoplasma genitalium*
NG: *Neisseria gonorrhoeae*
SAT: Simultaneous amplification and testing
PCR: Polymerase chain reaction
CIP: Ciprofloxacin
AZM: Azithromycin
CLR: Clarithromycin
ERY: Erythromycin
JOS: Josamycin
DOX: Doxycycline
OFX: Ofloxacin
PRI: Pristinamycin
TCY: Tetracycline
